# Manual Therapy Interventions in Patients With Chronic Obstructive Pulmonary Disease: A Comprehensive Narrative Review

**DOI:** 10.7759/cureus.62511

**Published:** 2024-06-17

**Authors:** Priyanka K Chilhate, Lajwanti Lalwani

**Affiliations:** 1 Department of Cardiovascular and Respiratory Physiotherapy, Ravi Nair Physiotherapy College, Datta Meghe Institute of Higher Education and Research, Wardha, IND

**Keywords:** pulmonary function, thoracic excursion, manual therapy, respiratory muscle, copd

## Abstract

Chronic obstructive pulmonary disease (COPD) is a breathing problem with ongoing airflow issues and changes in how the chest moves. Different manual therapy methods, like releasing the diaphragm, manipulating the spine and joints, and treating soft tissues, have been used for people with COPD. This review looks into how these manual therapy approaches affect COPD patients. Articles were searched in Google Scholar, PubMed, and Elsevier using keywords such as COPD, manual therapy, thoracic excursion, and pulmonary function. Only studies conducted between 2015 and 2023, employing randomized controlled trials (RCTs), crossover RCTs, or comparative studies with COPD subjects, thoracic excursion, chest expansion, or pulmonary function tests (PFTs) as outcome measures, and involving physiotherapy interventions were included. Out of 82 articles searched, 10 met the inclusion criteria, comprising six RCTs, three crossover RCTs, and one comparative study. Data extraction was performed by one reviewer, encompassing intervention descriptions, inclusion/exclusion criteria, baseline data, and outcome values. The findings suggest that conventional physiotherapy combined with manual therapy techniques such as stretching, osteopathic manual therapy, manual diaphragmatic release, soft tissue therapy, and spinal manipulation have improved thoracic excursion and pulmonary function in COPD patients. Therefore, these manual therapy techniques are recommended for enhancing thoracic excursion and pulmonary function in COPD patients.

## Introduction and background

Chronic obstructive pulmonary disease (COPD) is a respiratory disorder affecting small airways, leading to persistent airflow limitation, particularly during exhalation [1]. Synonymous medical terms for COPD include chronic airflow limitation disease, chronic obstructive airway disease, and chronic obstructive lung disease. COPD is a diverse lung condition with chronic respiratory symptoms like difficulty breathing, cough, and mucus production. It results from issues in the airways (bronchitis, bronchiolitis) and/or air sacs (emphysema), leading to ongoing and sometimes worsening airflow blockage. Two key signs of COPD are air getting trapped and the lungs becoming overly inflated [2].

Due to delayed diagnosis, COPD is one of the main causes of death and morbidity, with an underestimated prevalence rate [3]. To satisfy the body's increased metabolic demands, the respiratory muscles must continue to contract throughout an individual's life to maintain the proper level of ventilation [4]. Patients with COPD breathe in a paradoxical manner due to an increase in abdomen motion and a decrease in rib cage motion, due to the mechanical efficiency of their respiratory muscles [5].

Lengthening soft tissue surrounding the chest wall and respiratory muscles improves contraction force, lung volume, and breathing control. The diaphragm, being the primary respiratory muscle, undergoes modifications in its contractile force and influences lung volumes and capacities, when subjected to shortening. This phenomenon arises from considering lung volume as a matrix representing the length index of the respiratory muscle [6,7]. Stretching muscle fibers encourages the growth of sarcomeres, which in turn lengthens shortened muscles. A sufficient length of the respiratory muscles would encourage an overall improvement in their ability to contract, as well as a rise in thoracic expansion and benefits to respiratory mechanics performance [8,9].

In recent years, there has been increased interest in physical therapy as an additional therapeutic approach for COPD. Manual therapy includes a variety of hands-on treatments such as mobilization, manipulation, and soft tissue massage that aim to improve musculoskeletal function and promote general well-being. Manual therapy has typically been used to treat musculoskeletal disorders such as back pain and joint stiffness, but it is thought to offer potential benefits for COPD patients by focusing on thoracic mobility and respiratory muscle performance. The goal of this narrative review is to objectively analyze the available information on the efficacy of manual treatment on thoracic excursion and pulmonary function in COPD patients. By combining findings from relevant research, we seek to provide a complete review of the possible benefits and limitations of manual therapy as an additional treatment for COPD.

COPD is characterized by persistent airflow limitation and aberrant pulmonary inflammation resulting from prolonged exposure to noxious particles, notably from tobacco smoke. While inflammation of the lungs is common among smokers, individuals with COPD manifest an amplified reaction to harmful substances, culminating in chronic bronchitis, emphysema, and airway inflammation. These alterations lead to heightened airway resistance, reduced lung compliance, air entrapment, and advancing obstruction. The cellular and molecular pathways driving these pathological transformations in COPD are comprehensively elucidated [10].

Sustained exposure to noxious agents elicits the activation of innate defense mechanisms within the body. In the context of COPD, the immune response exhibits an aberrant sensitivity to harmful particulates and gases, notably those found in cigarette smoke. While all individuals who smoke encounter some degree of pulmonary inflammation, those afflicted with COPD manifest a disproportionate and dysregulated reaction, precipitating deleterious alterations. This hyperactive response may precipitate excessive mucous secretion, leading to the onset of chronic bronchitis characterized by persistent cough and heightened sputum production. Moreover, the structural integrity of lung tissue undergoes compromise, resulting in emphysematous changes typified by the degradation of parenchymal tissue, thereby diminishing elasticity and impairing gas exchange function. The disruption of physiological repair mechanisms and defense responses prompts inflammatory processes and fibrotic scarring within the confines of small airways, thereby exacerbating bronchiolar pathology [11].

These pathological modifications collectively culminate in heightened resistance within the smaller conducting airways, enhanced lung compliance, air entrapment, and advancing airflow obstruction-distinctive characteristics of COPD. The molecular and cellular underpinnings of these alterations entail a sequence of occurrences, encompassing oxidative stress, dysregulation in protease-antiprotease equilibrium, and persistent inflammatory processes. A profound comprehension of the intricate pathogenesis of COPD is imperative for the formulation of precision therapeutic strategies tailored to impede the progression of this incapacitating respiratory ailment [12].

In addition to difficulty breathing and a lasting cough, people with COPD often have trouble exercising. The main reasons for weakened respiratory muscles in COPD include changes in muscle fibers, low oxygen levels in the lungs and blood, high carbon dioxide levels, infections, poor nutrition, using corticosteroids for a long time, and not being physically active. Lung overexpansion makes it harder for the breathing muscles to work properly, so the chest tries to help by making the diaphragm muscle work less. This makes it tough to breathe as the extra breathing muscles have to work harder [13,14].

"Manual therapy" means using hands-on methods to work on soft tissues and joints. Skilled practitioners provide this type of treatment to enhance tissue flexibility, increase movement range, promote relaxation, and manage pain. It can also aid in repairing contractile and non-contractile tissues, improve stability or flexibility, lessen swelling and inflammation in soft tissues, remove restrictions, and make movement easier, enhancing overall function [15]. The basic idea behind using these mobility treatments for COPD patients is that they can temporarily enhance the flexibility of the chest wall. This is done by addressing a factor that causes stiffness in the chest wall [16]. Methods like myofascial release, soft tissue mobilization, stretching, massage therapy, high-velocity low amplitude thrust (HVLAT), and similar approaches are believed to encourage blood vessel widening and relaxation of smooth muscles. This, in turn, may enhance blood circulation, leading to improved flexibility, reduced pain, and positive changes in tissues [17].

## Review

Methodology

A comprehensive literature review was conducted using Google Scholar, PubMed, and Elsevier to identify articles related to COPD, respiratory muscles, manual therapy, thoracic excursion, and pulmonary function. The search focused on articles published between 2015 and 2023. Only randomized controlled trials (RCTs), crossover RCTs, and comparative studies that included subjects with COPD, and used thoracic excursion, chest expansion, or pulmonary function tests (PFTs) as outcome measures were considered. Physiotherapy interventions were required as part of the treatment. Excluded from the selection were articles categorized as correlational studies or case studies, those involving interventions other than physiotherapy or manual therapy, and studies conducted outside the specified time frame. Following the search, 82 articles were identified using Google Scholar, PubMed, and Elsevier, with 10 meeting the inclusion criteria for the study. For quality evaluation purposes, physiotherapists extracted comprehensive data, including intervention details, criteria for inclusion/exclusion, baseline information, and outcome values at baseline, post-intervention, and during follow-up.

Review

In a 2023 RCT conducted by Tsui et al., involving 30 participants, the study assessed the effects of chest wall mobilizations, which included stretching and joint mobilization, on individuals diagnosed with COPD. The intervention aimed to evaluate its impact on pulmonary function, respiratory muscle strength, thoracic excursion, and cervical/thoracic range of motion. Results indicated that integrating additional chest wall mobilization into COPD rehabilitation improved thoracic extension and rotation, ultimately enhancing lower thoracic excursion. This improvement in chest expansion capacity optimized the functioning of respiratory muscles, particularly beneficial for severe COPD cases, leading to increased respiratory muscle strength [18].

In a 2021 study by Swathi et al., with 60 participants, Group A (30 patients) did diaphragmatic stretching, and Group B (30 patients) did rib stretching. The study looked at lung function tests and chest movement. Results showed that diaphragmatic stretching was better than rib stretching for improving both lung function and chest movement in people with COPD [8].

In a 2020 study by Rehman et al., with 30 participants, one group received a hot pack followed by stretching of breathing muscles and gentle shoulder movements, while another group only had gentle shoulder movements after the hot pack. The study examined chest expansion, and the distance walked in six minutes. Results showed that adding breathing muscle stretching helped improve chest expansion and walking distance, suggesting it could be helpful for patients [19].

In a 2020 study by Ganesh et al., with 26 participants, two groups were tested. The first group, the HVLAT group, involved lying down or sitting comfortably while the therapist manipulated the upper, mid, and lower thorax. The second group, the exercise group, had participants sitting comfortably and doing breathing exercises and thoracic expansion exercises. The study measured lung function, chest and neck movement, and chest expansion. The results showed that neither intervention significantly affected breathing, movement, or chest expansion, except for flexibility in the middle back, neck bending, and sideways bending. Also, one treatment did not prove better than the other for immediate effects [13].

In a 2019 crossover RCT led by Maskey-Warzechowska et al., with 38 participants, two interventions were compared. The first was osteopathic manipulative treatment (OMT), which included various techniques like suboccipital decompression and thoracic lymphatic duct. The second was a sham treatment involving shoulder joint mobilization techniques. The study measured lung function tests and dyspnea levels using a visual analog scale. However, both OMT and sham procedures did not show significant differences in pulmonary function or dyspnea levels, indicating similar outcomes between the two interventions [20].

In a 2019 study by Nair et al., with 20 participants, they looked at two techniques to help people with COPD breathe better. The diaphragmatic stretch technique involved sitting, while the therapist gently pulled on the lower ribs during exhaling. The manual diaphragm release technique, done lying down, involved manual contact with the lower ribs, adjusting during breathing. Both techniques were found to be safe and recommended for improving breathing in COPD patients by enhancing diaphragmatic excursion and chest expansion [21].

In a 2017 RCT led by Rofiqul, with 30 participants, diaphragmatic manipulation combined with costal manipulation showed more significant benefits on chest movement, lung function, and functional capacity in individuals with COPD when compared to conventional treatment [22].

In a 2016 RCT by Rekha K et al., involving 30 participants, the experimental group treated with respiratory accessory muscle stretching and coordinated breathing exercises showed significant improvements in chest expansion, reduced dyspnea, and increased exercise tolerance compared to the control group receiving conventional methods, including diaphragmatic breathing exercises and thoracic mobility exercises [23].

In a 2016 study led by Engel et al., involving 33 participants, three distinct treatment protocols were implemented for pulmonary rehabilitation. Group 1 underwent the conventional rehabilitation program, whereas Group 2 received supplementary soft tissue therapy in conjunction with the standard regimen. Group 3 underwent a combined intervention involving soft tissue therapy and spinal manipulation alongside the standard program. The study comprehensively assessed various parameters, including PFTs, the six-minute walk distance (6MWD) test, responses to respiratory questionnaires, levels of anxiety and depression, and blood pressure measurements. Significantly, a notable improvement in forced vital capacity was evident among participants receiving both soft tissue therapy and spinal manipulation. Critically, no major or moderate adverse events were recorded throughout the intervention period [16].

In a 2015 study, Abdelaal et al. conducted an RCT involving a total of 195 participants. Subjects were randomly assigned to one of four groups: Group A (n = 46) received diaphragmatic manipulation, Group B (n = 53) received rib rising, Group C (n = 50) received a combination of diaphragmatic manipulation and rib rising, while Group D (n = 46) served as the control group. The primary outcomes measured were PFTs and the 6MWD. Results indicated that employing diaphragmatic and costal manipulative techniques demonstrated efficacy as therapeutic interventions for improving ventilatory function and functional capacity among individuals with COPD, with particularly notable benefits observed when these techniques were combined [24].

**Figure 1:**
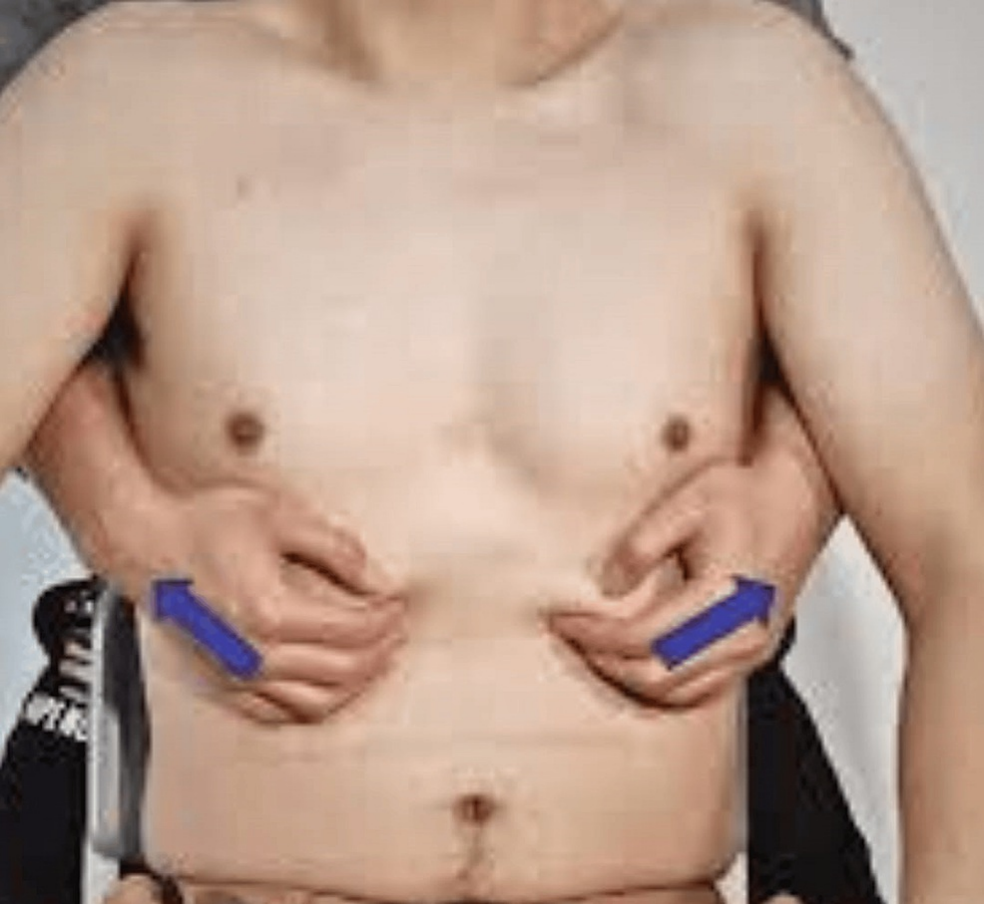
Diaphragmatic stretching Image credit: [25]

**Figure 2:**
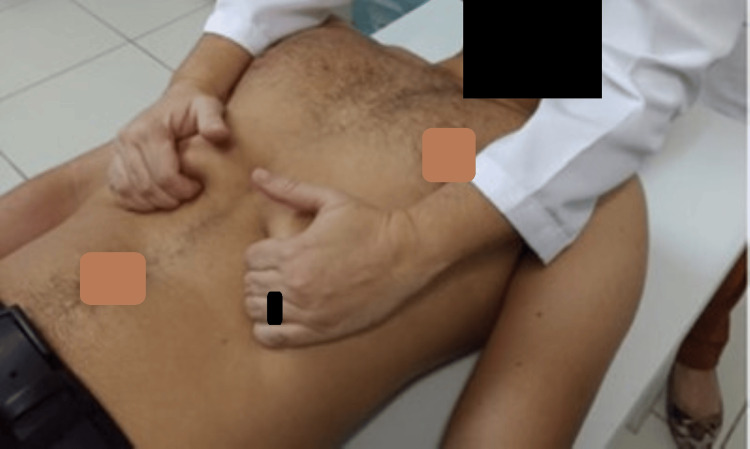
Manual diaphragm release technique Image credit: [26]

**Figure 3:**
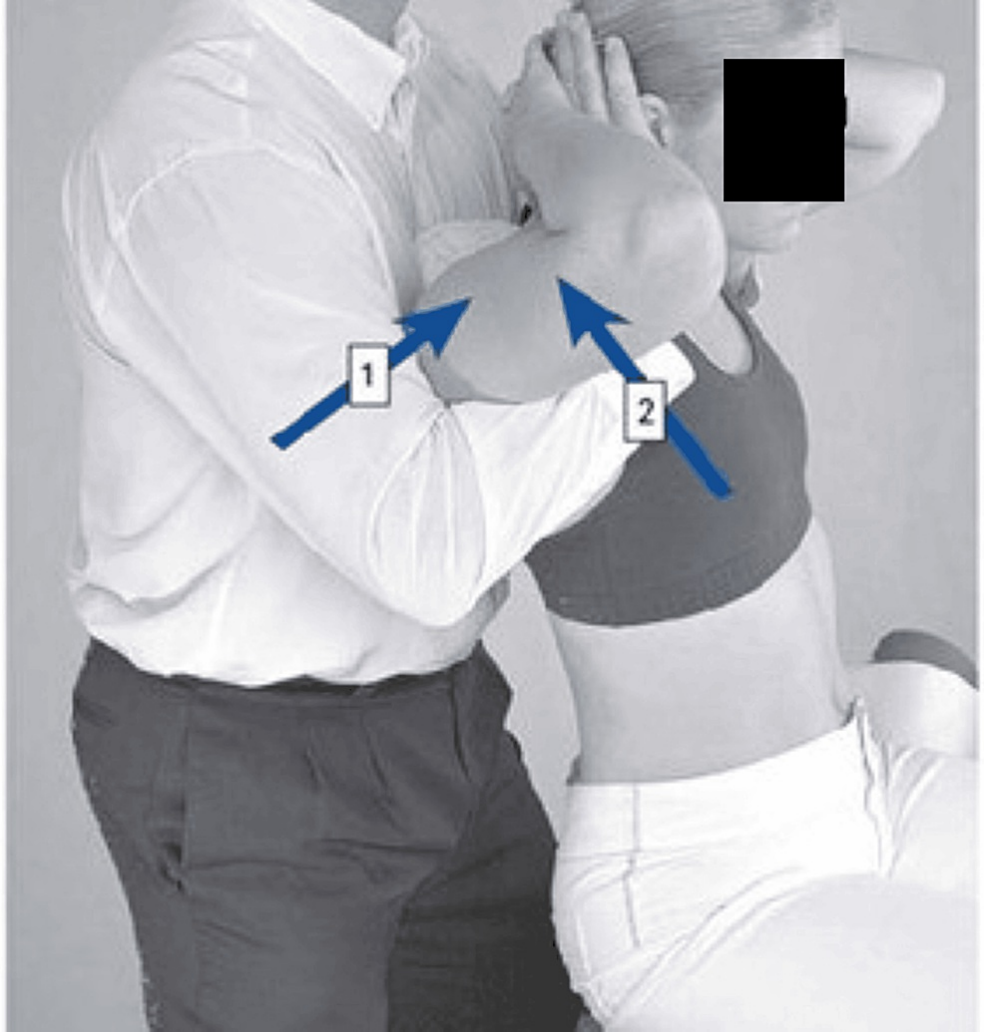
Spinal and rib manipulation Image credit: [16] 1) Posrerior-anterior force; 2) Anterior-posterior force

**Figure 4:**
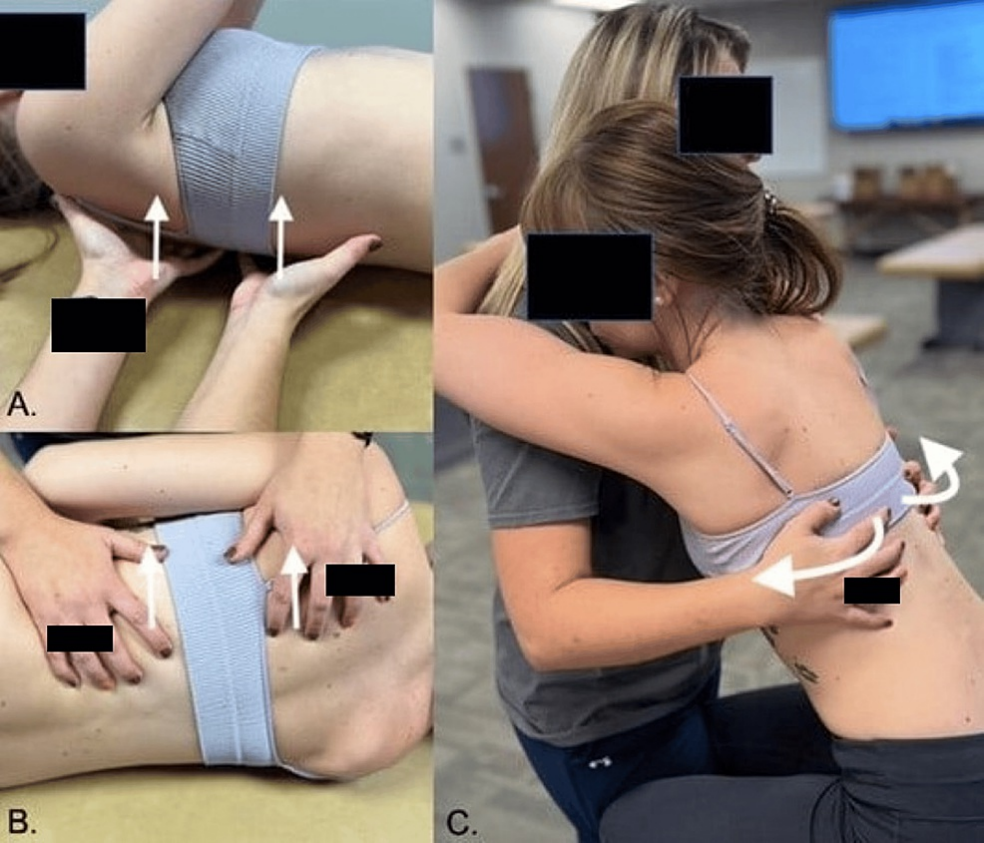
Rib-raising technique variations Image credit: [27] A) Supine position; B) Lateral recumbent positioning; C) Seated positioning

**Figure 5:**
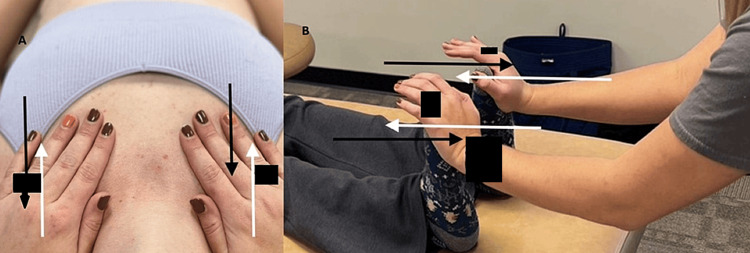
Lymphatic pump drainage Image credit: [27] A) Thoracic lymphatic drainage; B) Peripheral lymphatic drainage

Table 1 presents a narrative review made from evidence of clinical trials, indicating the impact of manual treatment on lung function and chest mobility in COPD patients. Figures 1-5 show techniques used in COPD patients for treatment.

**Table 1:** Evidence from clinical studies about how manual therapy affects chest movement and lung function in people with COPD COPD: Chronic obstructive pulmonary disease; PFT: Pulmonary function test; ROM: Range of motion; 6MWD: 6-minute walk distance; VAS: Visual analog scale; FEV1: Forced expiratory volume in 1 second; FVC: Forced vital capacity; SGRQ: St George's respiratory questionnaire; HADS: Hospital anxiety and depression scale; PR: Pulmonary rehabilitation; ST: Soft tissue; AEs: Adverse events; SM: Spinal manipulation; N: Number of participants; n: Number of participants in each group

Author	Design	Participants	Intervention	Outcome	Conclusion
Tsui et al. [18]	RCT	N = 30	Mobilizations of the chest wall incorporating both joint mobilization and stretching techniques.	PFT, respiratory muscle strength, thoracic excursion, cervical, and thoracic ROM	In patients with COPD, making the chest wall more flexible during rehab can help them breathe better. This is because it allows their lungs to expand more easily. For people with severe COPD, this can boost their breathing muscles, making them stronger.
Swathi et al. [8]	Comparative study	N = 60	Group A consisted of 30 patients who received training in diaphragmatic stretching, whereas Group B comprised 30 patients who received training in rib stretching.	PFT, thoracic excursion	Diaphragmatic stretching exercises demonstrate superior efficacy compared to rib stretching exercises in augmenting pulmonary function and thoracic mobility among individuals diagnosed with COPD.
Rehman et al. [19]	Comparative study	N = 30	The experimental group underwent a protocol involving the application of a hot pack followed by respiratory muscle stretching and gentle passive mobilization of the shoulder joints. In contrast, the control group received a hot pack followed by passive shoulder joint movements in a relaxed state.	Chest expansion, 6MWD	Improving patients' clinical condition, especially regarding chest expansion and performance in a six-minute walk test, can be accomplished through passive stretching of respiratory muscles.
Ganesh et al. [13]	RCT	N = 26	HVLAT group: Participants adopt a comfortable lying position with arms crossed, while the therapist stands on the opposite side. Manipulation targets specific thoracic vertebrae, utilizing a pistol grip hand position and guiding deep breathing to apply upward force. Exercise group: Participants sit comfortably, place their hands on their abdomen, and engage in pursed lip breathing along with thoracic expansion exercises targeting the upper, middle, and lower thoracic regions. The exercises consist of 10 repetitions and three sets.	PFT, ROM of thorax and cervical region Chest expansion	The research results indicated that neither intervention yielded significant alterations in respiratory parameters, range of motion, or chest expansion, except for enhancements in thoracolumbar flexibility, cervical flexion, and lateral flexion. No apparent superiority between the treatments was noted concerning their immediate effects.
Maskey-Warzechowska et al. [20]	Crossover RCT	N = 38	OMT: Techniques such as releasing tension at the base of the skull, loosening tight neck muscles, massaging the chest to help with lymph flow, and stretching the diaphragm were used, following Noll et al.'s method. Sham: Moving the shoulder in different directions using gentle techniques, easing tension in shoulder muscles and the biceps after muscle tightening. These methods were adapted from Noll et al.'s approach.	PFT, dyspnea (VAS)	There were no significant differences observed in pulmonary function or dyspnea before and after both OMT and sham procedures.
Nair et al. [21]	Crossover RCT	N = 20	Diaphragmatic stretch technique: Patients sit upright, and the therapist stands behind them, placing hands around the chest just below the ribs. The patient leans forward slightly to relax the stomach muscles. As the patient exhales, the therapist gently pulls down on the lower ribs, maintaining a firm but gentle grip while the patient inhales. Manual diaphragm release technique: The patient lies on their back with arms and legs relaxed. The therapist stands at the head and uses the lower part of their hand and last three fingers to touch below the ribs on both sides. With arms aligned towards the patient's shoulders, during inhalation, the therapist gently pulls the contact points towards the head and slightly to the side, following the rising ribs. During exhalation, the touch is shifted deeper towards the inner edge of the ribs, maintaining some resistance. The therapist repeats this process with each breath cycle.	Diaphragmatic excursion, chest expansion	The diaphragmatic stretch technique and manual diaphragm release technique can be safely recommended for patients with clinically stable COPD to improve diaphragmatic excursion and chest expansion.
Rofiqul [22]	RCT	N = 30	The experimental group received diaphragmatic manipulation, while the other group underwent conventional treatment.	PFT, 6MWD	Combining diaphragmatic manipulation with costal manipulation yields greater benefits in chest kinematics, lung function, and functional capacity for patients with COPD.
Rekha et al. [23]	RCT	N = 30	The experimental group received stretch and relax techniques targeting respiratory accessory muscles along with coordinated breathing exercises. In the hold phase, patients were instructed to breathe in, and during the relaxing phase, they were asked to breathe out. The control group underwent traditional treatment methods, involving diaphragmatic breathing exercises and exercises to enhance thoracic mobility.	Chest expansion, modified Borg’s scale, 6MWD	This study revealed that stretching respiratory accessory muscles significantly enhanced chest expansion, alleviated shortness of breath, and increased exercise tolerance levels in patients with COPD.
Engel et al. [16]	RCT	N = 33	Group 1 (PR) followed the usual pulmonary rehabilitation program. Group 2 (ST + PR) had soft tissue therapy alongside the standard PR program. Group 3 (ST + SM + PR) received a mix of soft tissue therapy, spinal manipulation, and the standard PR program.	PFT (FEV1, FVC), 6MWT, SGRQ, HAD scale, systolic and diastolic blood pressure	After 24 weeks, there was a significant difference in FVC among the three groups (p < 0.04). In the ST + SM + PR group, compared to the PR-only group, there was a meaningful increase of 0.40 liters (CI: 0.02, 0.79; p < 0.03). Importantly, no major or moderate AEs were reported after 131 ST and 272 SM sessions.
Abdelaal et al. [24]	RCT	N = 195	Participants were assigned randomly to four groups: diaphragmatic manipulation (Group A; n = 46), rib rising (Group B; n = 53), diaphragmatic manipulation plus rib rising (Group C; n = 50), and the control group (Group D; n = 46).	PFT, 6 MWD	Utilizing diaphragmatic and costal manipulative procedures proves effective as therapeutic interventions for enhancing ventilatory function and functional capacity in COPD patients, particularly when applied in combination.

## Conclusions

These articles report that patients with COPD have better thoracic excursion and pulmonary function when receiving conventional physiotherapy in addition to manual therapy techniques like stretching, osteopathic manual therapy, manual diaphragmatic release technique, soft tissue therapy, and spinal manipulation. Thus, these are a few manual therapy methods that are suggested for enhancing thoracic excursion and lung function in COPD patients. Research indicates that manual therapy, specifically for individuals with COPD, can enhance respiratory function by targeting the respiratory muscles. Ribcage contraction and extension enhance thoracic excursion and mobility, which in turn permits increased lung ventilation and expansion, ultimately leading to an improvement in respiratory function. Stretching exercises for the respiratory muscle can reduce stress, enhance muscular strength and coordination, and facilitate better breathing. In the future, it is crucial to conduct high-quality research that comprehensively evaluates the effects of manual therapy. Practitioners utilizing manual therapy should exercise clinical judgment alongside the insights provided by this review.
